# Clinically relevant pharmacokinetic knowledge on antibiotic dosing among intensive care professionals is insufficient: a cross-sectional study

**DOI:** 10.1186/s13054-019-2438-1

**Published:** 2019-05-22

**Authors:** Lucas M. Fleuren, Luca F. Roggeveen, Tingjie Guo, Petr Waldauf, Peter H. J. van der Voort, Rob J. Bosman, Eleonora L. Swart, Armand R. J. Girbes, Paul W. G. Elbers

**Affiliations:** 1 Department of Intensive Care Medicine, Research VUmc Intensive Care (REVIVE), Amsterdam Medical Data Science (AMDS), Amsterdam Cardiovascular Sciences (ACS), Amsterdam Infection and Immunity Institute (AI&II), Amsterdam UMC, Location VUmc, Vrije Universiteit Amsterdam, Amsterdam, The Netherlands; 2 Department of Anaesthesia and Intensive Care Medicine, Kralovske Vinohrady University Hospital and The Third Faculty of Medicine, Charles University, Prague, Czech Republic; 3 Intensive Care, OLVG, Amsterdam, The Netherlands; 4 Department of Clinical Pharmacology and Pharmacy, Amsterdam UMC, Location VUmc, Vrije Universiteit Amsterdam, Amsterdam, The Netherlands

**Keywords:** Antibiotics, Intensive care, Pharmacokinetics, Drug dosing

## Abstract

**Background:**

Antibiotic exposure in intensive care patients with sepsis is frequently inadequate and is associated with poorer outcomes. Antibiotic dosing is challenging in the intensive care, as critically ill patients have altered and fluctuating antibiotic pharmacokinetics that make current one-size-fits-all regimens unsatisfactory. Real-time bedside dosing software is not available yet, and therapeutic drug monitoring is typically used for few antibiotic classes and only allows for delayed dosing adaptation. Thus, adequate and timely antibiotic dosing continues to rely largely on the level of pharmacokinetic expertise in the ICU. Therefore, we set out to assess the level of knowledge on antibiotic pharmacokinetics among these intensive care professionals.

**Methods:**

In May 2018, we carried out a cross-sectional study by sending out an online survey on antibiotic dosing to more than 20,000 intensive care professionals. Questions were designed to cover relevant topics in pharmacokinetics related to intensive care antibiotic dosing. The preliminary pass mark was set by members of the examination committee for the European Diploma of Intensive Care using a modified Angoff approach. The final pass mark was corrected for clinical relevance as assessed for each question by international experts on pharmacokinetics.

**Results:**

A total of 1448 respondents completed the survey. Most of the respondents were intensivists (927 respondents, 64%) from 97 countries. Nearly all questions were considered clinically relevant by pharmacokinetic experts. The pass mark corrected for clinical relevance was 52.8 out of 93.7 points. Pass rates were 42.5% for intensivists, 36.1% for fellows, 24.8% for residents, and 5.8% for nurses. Scores without correction for clinical relevance were worse, indicating that respondents perform better on more relevant topics. Correct answers and concise clinical background are provided.

**Conclusions:**

Clinically relevant pharmacokinetic knowledge on antibiotic dosing among intensive care professionals is insufficient. This should be addressed given the importance of adequate antibiotic exposure in critically ill patients with sepsis. Solutions include improved education, intensified pharmacy support, therapeutic drug monitoring, or the use of real-time bedside dosing software. Questions may provide useful for teaching purposes.

**Electronic supplementary material:**

The online version of this article (10.1186/s13054-019-2438-1) contains supplementary material, which is available to authorized users.

## Background

Sepsis and septic shock remain one of the deadliest diseases in intensive care units worldwide [1, 2] and are estimated to contribute to more than one third of all hospital deaths [3]. Despite the magnitude of the sepsis burden, efforts to develop new treatments have been largely unsuccessful [4, 5]. Therefore, sepsis management continues to rely on source control, supportive measures, and adequate antibiotic treatment. This includes adequate antibiotic dosing to prevent toxicity and inadequate exposure.

Despite the importance of antibiotic dosing, antibiotic exposure is well known to be frequently inadequate in intensive care patients [6–8]. The DALI study showed that less than 50% of patients treated for infection with β-lactam antibiotics achieved their preferred pharmacokinetic target [8]. Similar observations have been reported in other studies for beta-lactam antibiotics [9, 10], as well as for fluoroquinolones [11, 12]. More importantly, the DALI study showed aberrant serum concentrations were associated with poorer outcome in clinical patients [8]. The rationale that underdosing leads to ineffective pathogen eradication seems probable for other antibiotics as well.

Admittedly, adequate antibiotic dosing for critically ill patients is challenging. Intensive care patients have markedly altered and variable pharmacokinetic parameters for antibiotics as compared to healthy individuals or less severely ill patients. Organ dysfunction, abnormal fluid balances, altered hemodynamics, and organ replacement therapy can severely impact dosing requirements [13]. However, guidelines fail to provide recommendations on dose adaptation or dose personalization in these patients [14]. Therefore, prescribing antibiotics routinely follows a one-size-fits-all principle.

Therapeutic drug monitoring may provide guidance, but is usually only provided for aminoglycosides [15] and the glycopeptide vancomycin [16], but not for other antibiotics. In addition, this guidance requires drug sampling and can therefore not be used at the start of antibiotic treatment, paradoxically when adequate dosing may be most important. Fully automated systems that provide real-time bedside advice and are integrated with the electronic patient record could be a solution, but require further development and clinical validation [17].

As a consequence, adequate pharmacokinetic knowledge remains pivotal to optimize antibiotic dosing at the bedside of the critically ill. It is currently not known whether the level of knowledge on pharmacometric principles among intensive care professionals is sufficient. Our hypothesis was that there is room for improvement given the signals of frequent inadequate antibiotic exposure in the critically ill. To test our hypothesis, we set out to assess the level of clinically relevant knowledge on pharmacokinetic principles governing antibiotic dosing in the setting of intensive care medicine using an expert-validated questionnaire. As a corollary, this questionnaire may serve as a validated educational tool. Therefore, we encourage readers to take the questionnaire themselves.

## Methods

In May 2018, we set up a cross-sectional study by sending out a questionnaire testing the level of knowledge on pharmacometric principles governing antibiotic dosing in the critically ill by electronic mail. Approximately 20,000 healthcare professionals in the field of intensive care medicine were approached using the professional networks of the authors and the database of the international fluid academy (iFAD) days meeting. iFAD comprises of an international collaboration group with the aim of improving outcomes in the critically ill through fluid management, organ support, and monitoring. Attendees include nurses and critical care specialists.

### Population

Target populations for the questionnaires were intensivists, residents, fellows, and intensive care nurses. No patients were involved in this study. For the purpose of this study, intensivist was defined as a medical specialist in critical care medicine. Fellows were defined as physicians with a dedicated program towards national or international accreditation as an intensivist. Residents were defined as all other junior physicians working in intensive care medicine. As considerable differences in medical postgraduate programs exist among countries, respondents themselves were asked to select the category most appropriate to them [18–21].

### Privacy and consent

Only individuals who consented to receive electronic mail related to intensive care medicine were approached. Recipients were asked to further disseminate the questionnaire in their professional network at their own discretion to yield more responses. All intensive care professionals that chose to respond provided written informed consent for use of their data, in compliance with the General Data Protection Regulation [22]. Participation was anonymous, and internet protocol addresses were not stored. We did collect additional data including date, time, and duration of questionnaire completion, age, years of work experience, hospital, and country. For privacy reasons, data on age and work experience were collected in discretized form using brackets and participants were not obliged to provide information on hospital and country.

### Questions

All questions were specifically developed for this questionnaire and designed to cover the clinically relevant topics related to antibiotic pharmacokinetics in the setting of intensive care medicine. The core competencies defined by the Competency-Based Training in Intensive Care Medicine in Europe (CoBaTrICE) collaboration provided a reference standard for these topics [23]. This yielded 12 questions, which can be found in Table 1 together with the answer key. The relationship between the questions and the CoBaTrICE collaboration and a review by Roberts et al. [13] can be found in the Additional file 1. Underlying principles and concise background for these questions can be found in Box 1. The maximum number of possible answers varied, and questions with multiple answers were allowed. No open questions were used for ease of automated scoring. Participants were asked to refrain from using other resources to fill out the questions and were asked not to discuss questions with colleagues that had not yet participated in the survey. The questionnaire contained a control question to verify whether additional sources such as colleagues, textbooks, or the Internet were used.

**Table 1:** Characteristics of the respondents

	Nurse	Resident	Fellow	Intensivist	Total
Respondents (#)	154	198	169	927	1448
Age (mean, yrs)	41.1	33.1	38.3	44.6	42
Work Experience (mean, yrs)	10.7	2.9	5.3	11.1	9.3
Time to completion (mean, min)	14.9	12.3	13.2	13.8	13.6
From European countries (#)	147	192	165	896	1400

*yrs* years, *min* minutes

### Modified Angoff scoring

Questions answered correctly resulted in 10 points; subquestions yielded part of the points amounting to a total of 10. To set the pass mark for the designed questions, we used a modified Angoff approach [24]. In this approach, subject matter experts each attribute a minimally competent candidate (MCC) score to each of the questions. This score represents the percentage of borderline candidates (i.e., those candidates that the subject matter expert expects to just have passed the exam) that would answer these individual questions correctly. This score was corrected for guessing and adjusted to acknowledge a theoretical maximum score using the formula: corrected score = subject matter expert score × (90 − score expected by guessing) + score expected by guessing. Finally, the corrected scores for individual questions are averaged to yield the exam Angoff score. The score has been used extensively in medical education with good reliability [25–27]. It has been shown that a second round of decision-making adds little to precision; this step was therefore omitted [28]. We chose members of the examination committee of the European Society of Intensive Care Medicine as our subject matter experts. Nine of these independently scored our questions. They are all experienced intensivists and responsible for the European Diploma in Intensive Care (EDIC) exams. In addition, they have ample experience in Angoff scoring. Therefore, our final pass mark can be seen as the level of knowledge that is expected from intensivists in independent practice.

### Assessment of clinical relevance

As an additional step, we asked pharmacokinetic experts to rate our questions on clinical relevance. A PubMed ReMiner-search was conducted to identify the top 10 publishing experts in intensive care pharmacokinetics [29]. Titles and abstracts were searched (“antibiotics,” “intensive care,” “pharmacokinetics”) and sorted per author, which resulted in a list of top publishing institutions. Among these, one author from each institution was selected, which yielded six authors. The scores for these six experts were averaged and served as a correction factor for the Angoff scores. The average of all clinically relevance-corrected Angoff scores for each question formed the pass mark for the survey. All analyses were performed using Python (Python Software Foundation. Python Language Reference, version 3.6.4).

## Results

A total of 1448 respondents completed the survey. Characteristics of the respondents are shown in Table 1. Most of the respondents were intensivists (*n* = 927, 64%); the majority of whom were between 40 and 50 years of age (*n* = 383, 41%). Most of the fellows (*n* = 117, 69%) were in their thirties, compared to 31% of intensivists. Experience in the practice of intensive care medicine varied widely, with nurses and intensivists having worked in their profession the longest. The largest group has worked in the ICU between 10 and 20 years (27% and 34% of nurses and intensivists respectively). Respondents from 97 different countries completed the survey, with the majority of those countries being located in Europe (74%). Even though completion of country of residence was not mandatory, it was provided in 1400 responses (97%).

**Fig. 1:**
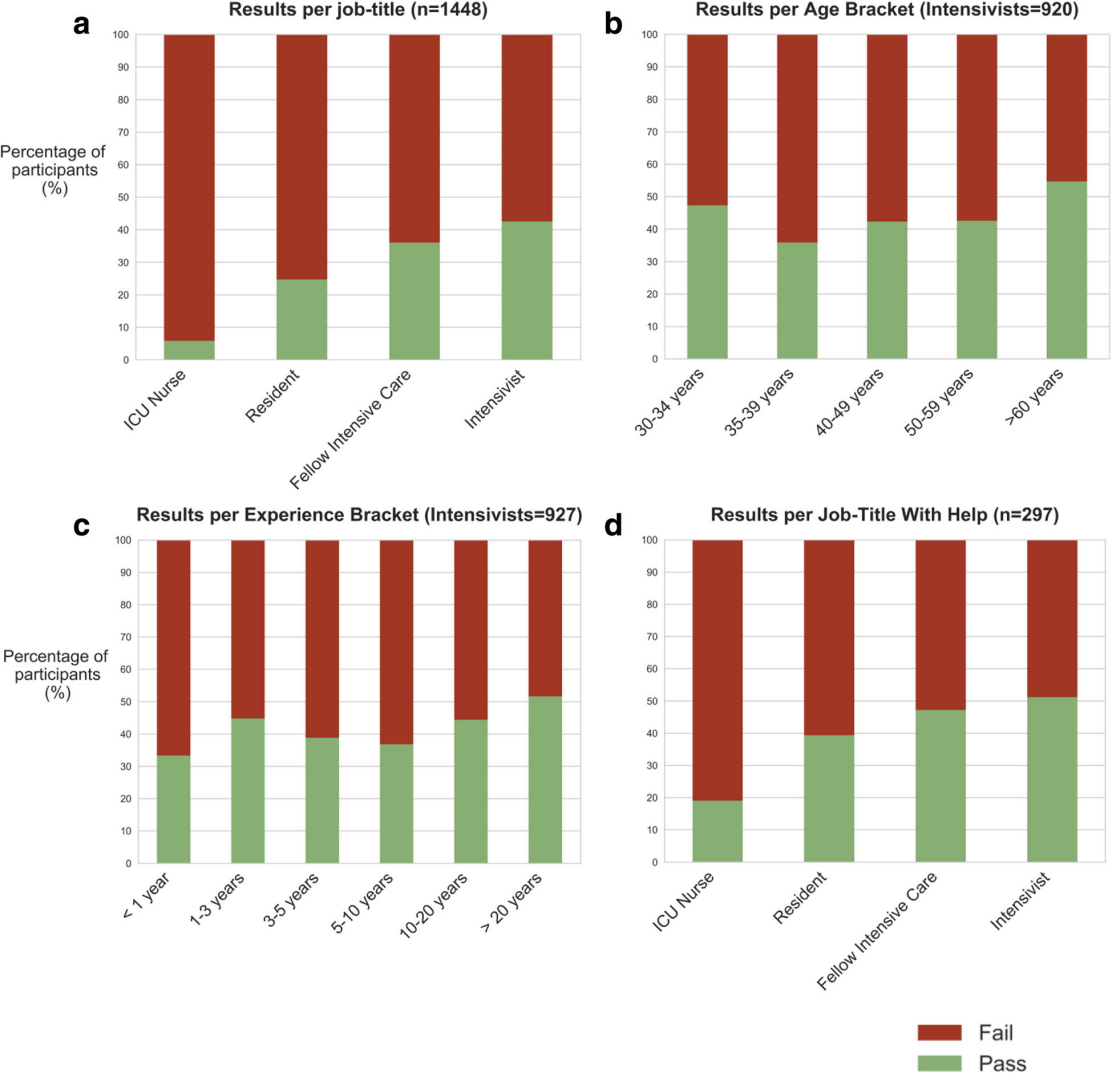
Scores of respondents. All results are percentages of respondents who passed based on the final pass mark adjusted for clinical relevance. **a** Survey results per job-title. **b** Intensivists’ scores per age bin. **c** Intensivists’ scores per years of experience, binned. **d** Results for respondents using additional resources to answer the questions

The Angoff pass mark was 70.8 out of 120 points (59.0% threshold). The final pass mark, adjusted for clinical relevancy, was 52.8 out of 93.7 points (56.4% threshold). Overall, 513 respondents (35.4%) passed with the final pass mark. Pass rates differed per job category; results are shown in Fig. 1a. Intensivists scored best (42.5%), followed by fellows (36.1%), residents (24.7%) and nurses (5.8%). Without correcting for clinical relevance, respondents scored lower (nurses 3.9%, residents 19.2%, fellows 20.1%, intensivists 30.1%). Two-hundred and ninety-seven respondents (21%) reported consulting books (50%), the Internet (88%), and colleagues (49%). Test results from the 297 respondents that used additional resources are shown in Fig. 1d. For fellows and intensivists, this led to an increase of more than 20% of respondents achieving the pass mark; for nurses and residents, absolute pass rates improved by 226% and 59%, respectively. Results from intensivists were stratified by age and years of ICU experience (results shown in Fig. 1b, c). No clear trend in the age group was observed, although intensivists with less than 1 year of experience tended to score lower. Percentage scores per country can be found in Additional file 1. Only countries with at least three respondents are shown (51 out of 97, 52.6%).

All survey questions with their model answers are shown in Table 2. Angoff scores, clinical relevance, and pass rates are shown for each question. Overall, clinical relevance is high for all questions, except for questions 10 (56/100) and 12 (49/100) on calculating half-lives. Angoff scores for some questions were below 60/100, indicating questions were hard. Both questions that have low clinical relevance also showed low Angoff scores. Intensivists’ pass rates per question range from 14.6 to 98.1%. Questions pertaining to Vancomycin excretion and antibiotic dosing in renal dysfunction showed high pass rates (90% and ≥ 90% for meropenem, ciprofloxacin, and ceftriaxone, respectively). Box 1 shows concise explanations for all questions.

**Table 2:** Questions and model answers with their respective Angoff scores and clinical relevance

	Question	Answer	Angoff score^a^	Clinical relevance^a^	Pass intensivists (%)
1	Are these antibiotics lipophilic or hydrophilic?				
	Vancomycin	Hydrophilic	64	74	60.2
	Ceftriaxone	Hydrophilic	64	74	44.6
	Meropenem	Hydrophilic	64	74	49.1
	Ciprofloxacin	Lipophilic	64	74	37.2
2	Which antibiotic is barely protein-bound?	Meropenem	42	71	14.6
3	For which antibiotic, using continuous infusion, is a loading dose least (!) important	Meropenem	49	83	18.3
4	In case of severe renal dysfunction, how should the maintenance dose be adapted for these antibiotics?				
	Vancomycin	Lower the dose^b^	74	94	98.2
	Ceftriaxone	Lower the dose^b^	68	94	96.0
	Meropenem	Lower the dose^b^	72	94	90.0
	Ciprofloxacin	Lower the dose^b^	70	94	70.4
5	In case of severe renal dysfunction, how should the initial dose be adapted for these antibiotics?				
	Vancomycin	No adaptation	75	93	65.5
	Ceftriaxone	No adaptation	72	93	85.0
	Meropenem	No adaptation	74	93	64.1
	Ciprofloxacin	No adaptation	74	93	66.9
6	Which treatment goal is most relevant for these antibiotics?				
	Vancomycin	AUC_0–24_/MIC	57	87	31.8
	Ceftriaxone	T > MIC	60	87	45.2
	Meropenem	T > MIC	60	87	49.9
	Ciprofloxacin	AUC0-24/MIC	54	87	31.6
7	How are these antibiotics cleared?				
	Vancomycin	Mostly renally	64	89	90.0
	Ceftriaxone	Both renally and via liver/bile/feces	59	89	31.0
	Meropenem	Mostly renally	60	89	48.8
	Ciprofloxacin	Both renally and via liver/bile/feces	60	89	30.7
8	What are risk factors for augmented renal clearance?				
	Cardiac arrest	False	66	87	82.1
	Prolonged ICU admittance	False	65	87	73.3
	Advanced age	False	65	87	78.8
	Multi-trauma	True	65	87	47.6
	Limited comorbidity	True	65	87	33.1
9	How do these parameters change in the initial phase of septic shock following adequate volume resuscitation?				
	Volume of distribution	Increases	61	85	87.1
	Clearance	Increases	61	85	35.5
10	The volume of distribution of an antibiotic is 100 L. Clearance is 10 L/h. What is the half-life?	About 7 h	50	56	41.6
11	What happens to half-life if …				
	Clearance increases	Decreases	64	70	88.9
	Clearance decreases	Increases	65	70	89.8
	Volume of distribution increases	Increases	63	70	40.5
	Volume of distribution decreases	Decreases	63	70	39.4
12	The half-life of an antibiotic is 3 h. When is steady state reached approximately following start of continuous infusion?	13–17 h	49	49	38.8

^a^Score out of 100

^b^Multiple answers can be correct; see Box 1

## Discussion

This is the first study to show that clinically relevant pharmacokinetic knowledge on antibiotic dosing among international intensive care professionals is insufficient. More than half of intensivists failed the test, while fellows, resident, and nurses had even lower scores. Thus, we have identified a major knowledge gap. Given the pivotal importance of adequate antibiotic dosing, this should be addressed.

The importance of pharmacokinetic principles to guide antibiotic dosing in critically ill patient is well recognized given the markedly altered and often changing pharmacokinetics in the critically ill [6, 8]. In particular, the DALI study showed that low antibiotic serum concentrations are associated with worse outcome in ICU patients [8]. Therefore, it is surprising that pharmacokinetic education does not have a prominent role in medical education, even though clinical educational tools are readily available [30]. The lack of pharmacokinetic expertise among intensive care professionals has likely contributed to the tolerance of standard dosing regimens for many antibiotics, even in the setting of intensive care medicine. This one-size-fits-all principle is also reflected in most national and international guidelines which fail to recommend individualized dosing strategies.

Education such as antimicrobial stewardship is a potential solution to improve pharmacokinetic expertise among intensive care professionals in order to optimize antibiotic dosing. However, large improvements in pharmacometric knowledge among intensive care professionals may not prove realistic. Causes include increasing workload in clinic [31] and the growing body of medical literature to stay up to date with [32]. An alternative solution could therefore be the extended use of therapeutic drug monitoring and increased support by clinical pharmacists and microbiologists. For vancomycin and the aminoglycosides, therapeutic drug monitoring has shown to increase efficacy and limit the occurrence of nephrotoxicity [15, 16]. Studies on therapeutic drug monitoring for the beta lactams are ongoing.

Automated pharmacokinetic modeling systems are another viable solution to tackle inadequate antibiotic exposure in the setting of intensive care medicine. The advent of electronic health record systems in most ICUs in resource-rich settings allows for continuous data feeds into integrated pharmacometric software, resulting in individual dosing recommendation at the bed side in real time [33, 34]. Other than therapeutic drug monitoring, these models could provide dosing advice based on the large amount of routinely collected clinical parameters rather than based on antibiotic samples alone. Advantages of these systems include immediate availability of dosing recommendations, i.e., even before the first dose, and the continuous correction of these recommendations at the touch of a button, based on a changing physiology in the critically ill. Evidently, safety and efficacy should be of unconditional importance when designing and implementing these systems. Therefore, such systems are currently still under investigation [17].

This study has several strengths. First, all questions were based on the Cobatrice framework, which ensures close adherence to validated training and examination goals for intensivists. Second, the number of respondents was high and their background was heterogeneous, which extends the results to an international audience of ICU professionals. Third, the pass mark and clinical relevance were assessed by members of the ESICM examination committee and world-renowned experts on pharmacokinetics, which assures test validity. Scores were adjusted for clinical relevance as an extra step after Angoff scoring. The two questions that were rated hard (i.e., lower Angoff scores) concomitantly had lower clinical relevance scoring, which was therefore adjusted for in the final scores.

This study also has some limitations. Firstly, although the number of responses is high, the response rate is low. We asked people to disperse the survey to colleagues to increase the number of respondents, which also clouds the response rate. We risk that only people who felt comfortable with the questions completed the survey. This would imply, however, that our score is an overestimation. Although we asked people to refrain from using other resources to answer the questions, 21% sought help, which would also contribute to the overestimation of their personal knowledge on the subject. Additionally, the number of respondents per country might not be a representative sample of that country. We therefore refrain from drawing conclusions on a per country basis. The sample from multiple countries, however, implies trends are similar in an international population. Lastly, definitions of intensive care units vary worldwide due to available resources and historical trends. Concomitantly, definitions of job titles in the ICU differ per region or even within countries [20, 21]. We assumed, however, that the title *intensivist* is reserved globally for doctors taking care of patients threatened in their vital parameters, including sepsis and septic shock.

## Conclusion

In conclusion, we showed that clinically relevant pharmacokinetic knowledge on antibiotic dosing among intensive care professionals is insufficient. This should be addressed, as suboptimal dosing strategies are associated with poorer outcomes. Options include extended use of therapeutic drug monitoring and pharmacist support as well as automated pharmacokinetics systems that provide dosing advice at the bedside in real time.

Box 1. Pharmacokinetic background Pharmacokinetics (the ancient Greek kinetikos meaning “putting in motion”) deals with drug movement *into* (absorption), *within* (distribution), and *out of* (metabolism and excretion) the body. All of these are subject to major alterations in critically ill patients, necessitating adaptations in antibiotic dosing. The following brief educational overview addresses relevant pharmacokinetic changes and provides explanations for the answers to our test questions. Changes in absorption are omitted here as antibiotics should always be given intravenously in critically ill patients (100% absorption).

**Distribution—antibiotic properties in the ICU (Q1, 2, 9, 11)**

The apparent volume of distribution (*V*_D_) of an antibiotic represents the necessary theoretical volume that contains the amount of administered drug to maintain the observed plasma concentration. *V*_D_ is derived by the amount of drug in the body/concentration measured in plasma. Size of antibiotic molecules, protein binding, and preference for aqueous (hydrophilic) or lipid (lipophilic) environments are properties of an antibiotic and influence antibiotic distribution and thus *V*_D_ (see Table 3). Additionally, in critically ill septic patients, capillary leak, fluid resuscitation, and inotrope administration may decrease antibiotic concentrations and therefore increase *V*_D_. *V*_D_ is related to elimination, as increases in *V*_D_ indicate a decrease in elimination rate or an increase in half-life, as half-life is defined as t1/2 = 0.693 × *V*_D_/CL. An increase in *V*_D_ may be thought of as a lower plasma concentration presenting to the kidneys, clearing the plasma from the drug.

**Table 3:** Properties of antibiotics. Results from multiple online resources [35, 36]

	V_D_ (L)	Lipo-/hydrophilic	T_1/2_ (h)	Protein binding	Renal clearance	Treatment goal
Vancomycin	32–68	Hydrophilic	5–11	55%	75–90%	AUC_0–24_/MIC
Ceftriaxone	7–12	Hydrophilic	8	85–95%	60%	*T* > MIC
Meropenem	11–27	Hydrophilic	1	2%	50–75%	*T* > MIC
Ciprofloxacin	150–225	Lipophilic	4–7	20–40%	75%	AUC_0–24_/MIC

**Metabolism—enzymatic function in the critically ill**

The liver metabolizes drugs through phase I (oxidation—CYP enzymes) and phase II (conjugation) reactions. During sepsis, hepatic dysfunction, hypo- and hyperthermia, and altered hepatic blood flow, among others, may influence drug metabolism [37]. The effect on antibiotic levels has not been completely elucidated.

**Elimination—routes and dosage adaptations (Q3, 5, 7, 8, 9, 10, 11, 12)**

Antibiotic elimination is mostly renal and to a lesser extent through hepatic routes, depending on antibiotic class. Hepatic elimination is generally related to cardiac output, which may be increased in sepsis. Likewise, augmented renal clearance may occur in the very early phase of critical illness, while impaired renal function is common at later stages [38, 39]. Risk factors for augmented renal clearance include young age, multi-trauma, and limited comorbidity, which could reflect the ability to recruit renal reserve [38, 39]. The time to reach steady state depends on half-life and thus is related to *V*_D_ and CL. Steady state is reached after 4 to 5 half-lives. This implies that for antibiotics with a long half-life, time to target concentration may be too long, necessitating a loading dose. This loading dose depends on *V*_D_ only and not on CL or rate of elimination. Therefore, in the setting of decreased clearance, e.g., because of renal failure, the loading dose should still be given in full. This can be thought of as the full *V*_D_ needed to be filled to quickly attain target concentration. An antibiotic with a low volume of distribution and short half-life such as meropenem will therefore quickly reach steady-state target concentration and requires no or low loading doses.

**Treatment goals and dosing (Q4, 6)**

Pre-clinical and clinical studies have identified pharmacokinetic treatment goals for antibiotics (Table 1). Depending on antibiotic class, these may be concentration (Cmax/MIC) or time (*T* > MIC) dependent, or a combination of both (AUC_0–24_/MIC) [13]. In case of reduced clearance, e.g., because of renal failure, time-dependent treatment goals require prolongation of the dosing interval as maximum concentration—C_max_—and *T* > MIC will remain unchanged. For AUC/MIC targets, the goal is to maintain AUC, possible through both a decrease in the maintenance dose and elongation of the interval. The smallest decrease in AUC is observed with a lowering of the dose and is therefore preferred. For some antibiotics, a decrease in renal clearance leads to an increase of hepatic clearance (ceftriaxone) in healthy subject, but this effect was not observed in a critically ill population [40].

## Additional file

Additional file 1: Result per country for intensivists (percentages and absolute numbers). (DOC 434 kb)
