# Supplementary material for: Clinically relevant pharmacokinetic knowledge on antibiotic dosing among intensive care professionals is insufficient: a cross-sectional study
Source: Crit Care. 2019 May 22;23:185. doi: 10.1186/s13054-019-2438-1 (PMC6532162; doi:10.1186/s13054-019-2438-1)
Supplement: Supplementary file 1 — Result per country for intensivists (percentages and absolute numbers). (DOC 434 kb) [file 13054_2019_2438_MOESM1_ESM.doc]

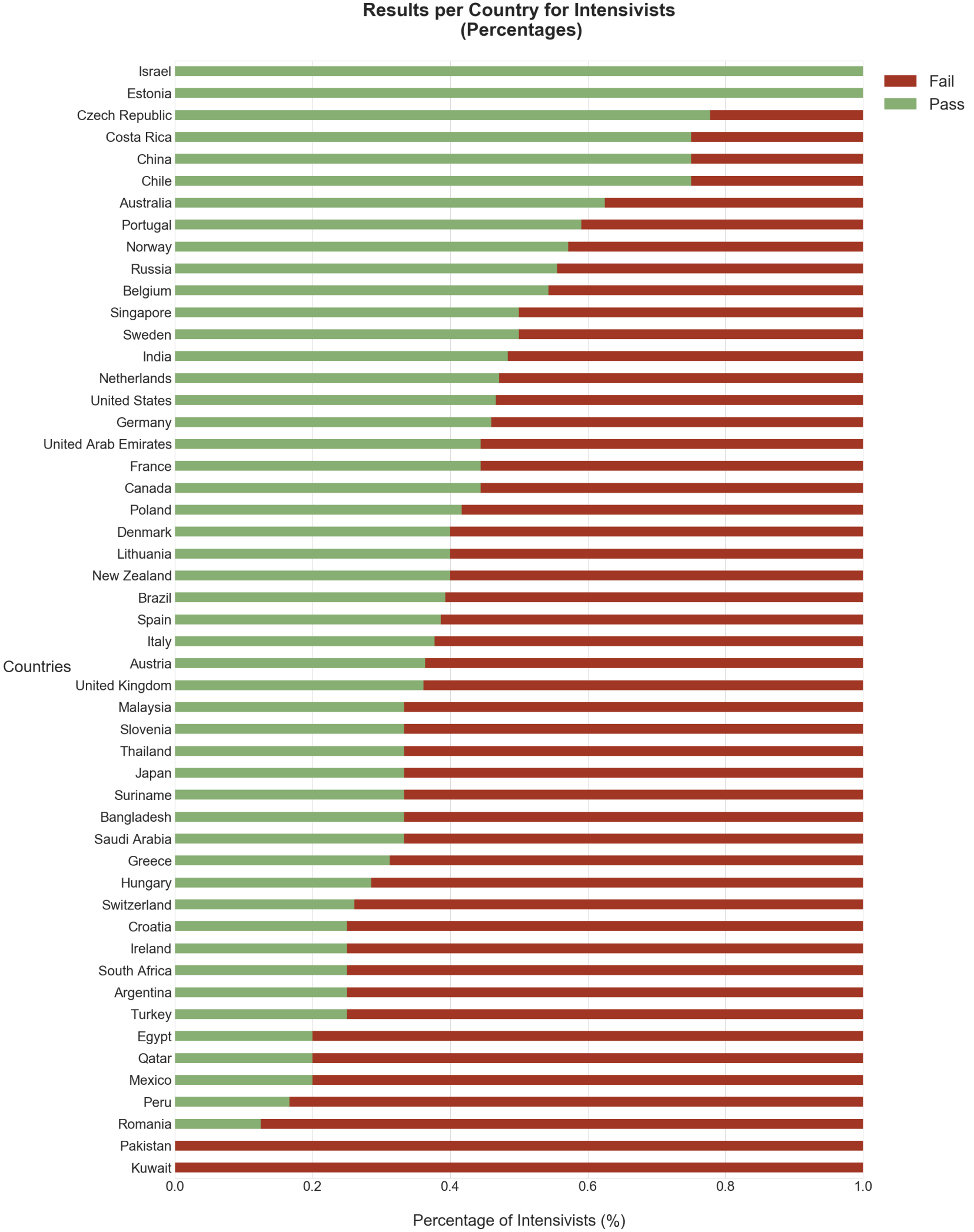
**Supplementary Material**

**Fig. 4** Result per country for intensivists (percentages). Only countries with at least three respondents are shown


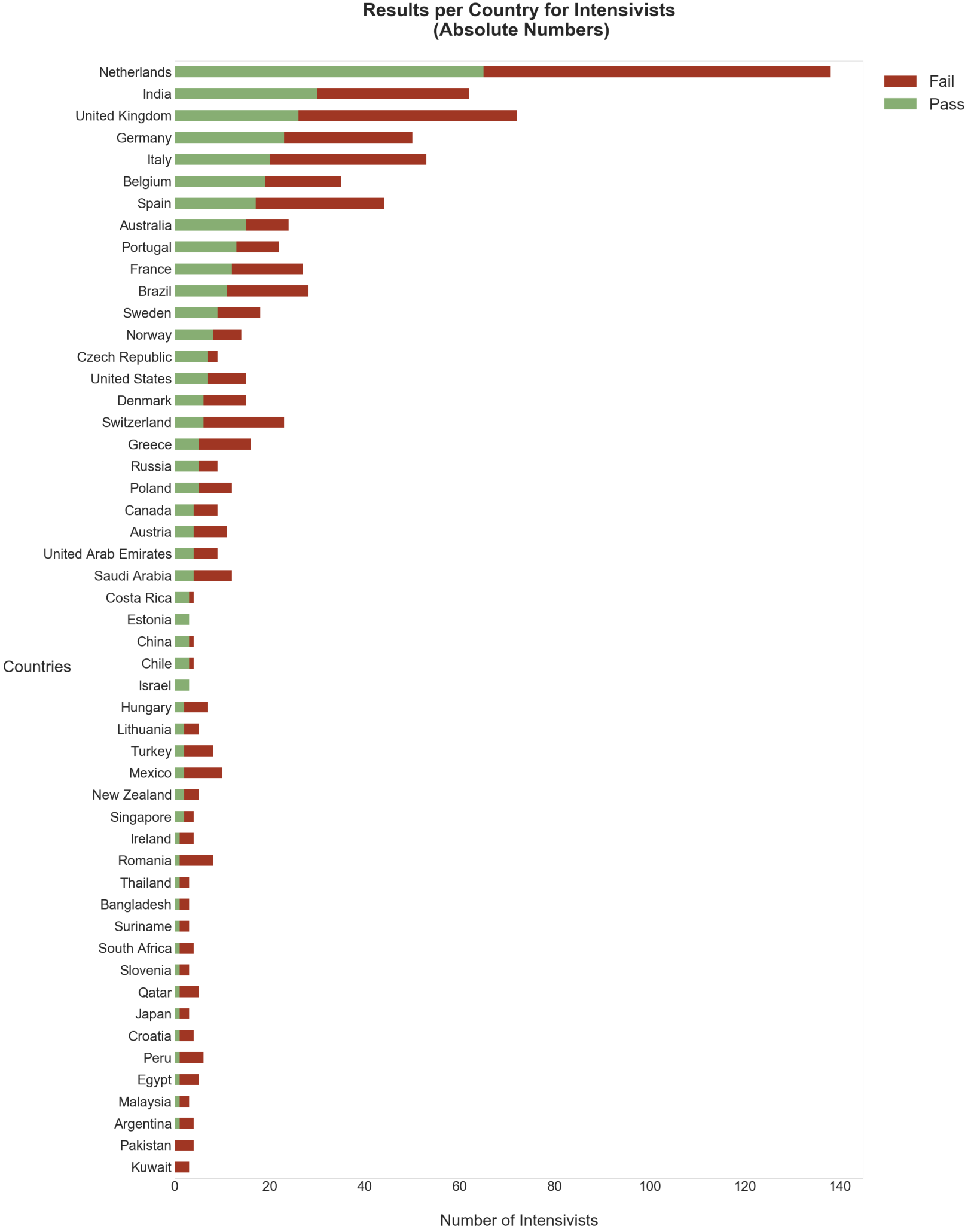


**Fig. 5** Result per country for intensivists (absolute numbers). Only countries with at least three respondents are shown

Table 1. Pharmacokinetic domains in critical illness identified by Roberts et al[1] in review and CoBaTrICE competencies[2], [3]

| **Table 1** | | | **Domains by CoBaTrICE** | | | | | |  |
| --- | --- | --- | --- | --- | --- | --- | --- | --- | --- |
| **Domains by Roberts et al** | | | *Factors determining the distribution of drugs* | *Distribution of drugs to organs and tissues* | *Modes of drug elimination* | *Pharmacokinetic analysis* | *Effects of acute organ failure (liver, kidney) on drug elimination* | *Influence of renal replacement therapies on clearance of commonly used drugs* | **Question** |
| *The effect of critical illness pathophysiology on pharmacokinetics* | | |  |  |  |  |  |  |  |
|  | *Cardiovascular system* | |  |  |  |  |  |  |  |
|  |  | *Fluid shifts, ‘third spacing’ and fluid overload* | x | x |  |  | x |  | 1, 9 |
|  |  | *Hypoalbuminaemia and altered protein concentrations* | x | x |  |  | x |  | 2 |
|  |  | *Tissue perfusion and target site distributions of antibiotics* | x | x |  |  | x |  | 3, 11, 12 |
|  | *The renal system* | |  |  |  |  |  |  |  |
|  |  | *Augmented renal clearance (ARC)* |  |  | x |  | x |  | 8 |
|  |  | *Renal dysfunction* |  |  | x | x | x | x | 4, 5, 7 |
|  |  | *Renal replacement therapy* |  |  | x |  | x | x | 1, 2, 10, 11 |
|  | *The pulmonary system* | | x | x |  |  | x |  | 1, 2 |
|  | *The hepatic system* | |  |  | x |  | x |  | 7 |
| *Increased incidence of reduced bacterial susceptibility* | | |  |  |  | x |  |  | 6 |

All statements listed in italic were directly retrieved from reference sources . Drug uptake, bioavailability, and drug formulation have been left out from the CoBaTrICE domains, as we assumed antibiotics in critically ill patients are administered intravenously.

References:

[1] J. A. Roberts *et al.*, “Review Individualised antibiotic dosing for patients who are critically ill: challenges and potential solutions,” *Lancet Infect. Dis.*, vol. 14, pp. 498–509, 2014.

[2] T. C. Collaboration, “Development of core competencies for an international training programme in intensive care medicine,” *Intensive Care Med.*, vol. 32, no. 9, pp. 1371–1383, Sep. 2006.

[3] J. Bion and H. U. Rothen, “CRITICAL CARE PERSPECTIVE Models for Intensive Care Training A European Perspective,” 2014.
